# Analysis of association between brain natriuretic peptide levels and blood pressure variability

**DOI:** 10.3892/etm.2014.1692

**Published:** 2014-04-25

**Authors:** HISASHI MASUGATA, SHOICHI SENDA, MICHIO INUKAI, TAKASHI HIMOTO, NAOHISA HOSOMI, HIROKI OKADA, FUMINORI GODA

**Affiliations:** 1 Department of Integrated Medicine, Kagawa University, Miki, Kagawa 761-0793, Japan; 2 Department of Clinical Neuroscience and Therapeutics, Hiroshima University Graduate School of Biomedical Sciences, Hiroshima 734-8551, Japan; 3 Department of Medical Education, Kagawa University, Miki, Kagawa 761-0793, Japan

**Keywords:** brain natriuretic peptide, hypertension, blood pressure variability

## Abstract

The present study aimed to investigate the association between plasma brain natriuretic peptide (BNP) levels and systolic blood pressure (SBP) variability over a one-year period. Blood pressure was measured in 44 patients treated for hypertension (73±9 years old) at an outpatient clinic every one to two months over a one-year period. The standard deviation (SD) and the coefficient of variation (CV) were calculated to assess SBP variability. Mean SBP was also calculated over the year. Plasma BNP levels were measured at the end of the one-year period. BNP was found to correlate with mean SBP (r=0.599; P<0.001). However, BNP was not observed to be correlate with either the SD (r=0.219; P=0.153) or the CV (r=0.058; P=0.709) of the SBP. Multiple regression analysis revealed that only the mean values of SBP were independently associated with BNP (β=0.613; P<0.001). Thus, BNP was found to be correlated with mean SBP, but not SBP variability. In conclusion, plasma BNP levels may reflect the average SBP, but not SBP variability over the one-year period prior to the measurement of BNP in patients with hypertension.

## Introduction

Brain natriuretic peptide (BNP), a hormone secreted by ventricular cardiomyocytes in response to pressure overload in the left ventricle (1), reflects the presence of left ventricular hypertrophy in patients with hypertension (2,3). Thus, BNP is used during antihypertensive treatment in order to assess hypertensive cardiac damage (4) and risk stratification (5). Previous studies (6,7) have demonstrated that antihypertensive treatment lowers plasma BNP levels; therefore, BNP levels may be used as a marker of blood pressure control. Therefore, it is hypothesized that may be an association between BNP and mean systolic blood pressure (SBP) during the antihypertensive treatment of patients with hypertension.

Seasonal variation in blood pressure has been reported (8–15), with blood pressure often higher during the winter than the summer. Previous studies in older adults (16,17) have revealed that a 10 mmHg rise in SBP is associated with an ~10% increase in the risk of mortality from stroke or ischemic heart disease. Thus, seasonal variation in SBP may influence mortality induced by cardiovascular events, including stroke and ischemic heart disease. Visit-to-visit variability in SBP has been established to be a strong predictor of stroke, independent of mean SBP (18,19). Visit-to-visit variability in SBP over a one-year period may reflect seasonal SBP variation. However, the association between seasonal SBP variation and plasma BNP levels in patients with hypertension is yet to be elucidated.

In the present study, it was hypothesized that SBP variability over a one-year period may reflect cardiac damage and may be significantly correlated with plasma BNP levels. In order to evaluate this hypothesis, the association between SBP variability over a one-year period and plasma BNP levels was assessed in patients with hypertension. This association was then compared with the association between mean SBP and BNP levels in these patients.

## Material and methods

### Subjects and protocol

A total of 44 patients [21 male and 23 female; mean age ± standard deviation (SD), 73±9 years; range, 53–87 years] who had been diagnosed with hypertension at Kagawa University Hospital (Kagawa, Japan), and who had visited the outpatient clinic regularly between September 2012 and August 2013, were included in the present study. Hypertension was defined as SBP ≥140 mmHg and/or diastolic blood pressure (DBP) ≥90 mmHg. Blood pressure was determined using the conventional cuff method. All patients were treated with at least one antihypertensive drug. For at least one year prior to the present study, as well as during the present study, the antihypertensive drug regimens of the patients did not change. Patients with a history of heart failure or evident heart disease were excluded and none of the study subjects had a history of atherosclerotic cardiovascular disease or stroke. Patient blood pressure was measured at an outpatient clinic every one to two months during the study year. At the end of the observation period, blood samples were taken in the morning after an 8-h overnight fast. Total cholesterol, triglycerides, high-density lipoprotein cholesterol, blood urea nitrogen, creatinine, uric acid, hemoglobin, C-reactive protein and glycosylated hemoglobin were measured using standard laboratory techniques. Patients with renal dysfunction whose creatinine levels were ≥1.4 mg/dl were excluded from the present study. The association between plasma BNP levels and various clinical parameters, including blood pressure and laboratory data, were analyzed. This protocol was approved by the Ethics Committee of Kagawa University (Kagawa, Japan). Informed consent was obtained from all participants.

### Blood pressure parameters

Patient SBP was measured in the morning at an outpatient clinic. Patients were not instructed as to whether to take their hypertension medication prior to or subsequent to the clinical visit. Upon arrival at the outpatient clinic, patients rested for ≥1 min and SBP was then measured. SBP was measured once, while the patient was seated, by a physician who was trained in using the automated oscillometric sphygmomanometer (HEM-1040; Omron, Kyoto, Japan) and blinded to patient blood examination data. The SD and the coefficient of variation (CV) of the SBP values obtained over the one-year observation period were calculated in order to assess SBP variability. The mean SBP values over the year were also calculated to assess average blood pressure control.

### Plasma BNP measurement

Plasma BNP levels were measured at the end of the observation period. In brief, samples were taken by a laboratory using an MI02 Shionogi BNP instrument (Shionogi & Co., Ltd., Osaka, Japan). Each blood sample was collected in an EDTA-2Na-treated blood sampling tube and then centrifuged at a low temperature to separate the plasma for the assay.

### Statistical analysis

Data are expressed as the mean ± SD. Statistical analyses were performed using SPSS software, version 18.0 (SPSS, Inc., Chicago, IL, USA). For the comparison of monthly mean values of SBP obtained from the patients over the study period, one-way analysis of variance (ANOVA) was used. Linear regression analysis was performed to analyze the association between plasma BNP levels, blood pressure and other variables. Step-wise multiple regression analysis was performed to identify which blood pressure parameters were independently associated with plasma BNP levels. A value of P<0.05 was considered to indicate statistical significance.

## Results

### Clinical characteristics of the subjects

The clinical parameters of the study subjects are summarized in Table I. The mean SBP (130±13 mmHg) and DBP (69±6 mmHg) values during the one-year observation were not particularly high, due to the antihypertensive treatments taken by the patients. However, the high mean plasma BNP values (39.0±25.7 pg/ml) indicated the presence of cardiac damage in this group of patients.

### SBP variability

The variation in SBP over the one-year observation period in all subjects is shown in Table II. The highest levels of SBP were observed in December (135±18 mmHg) and March (135±16 mmHg) and the lowest levels were observed in September (126±14 mmHg). However, no statistically significant differences were identified in the monthly mean values of SBP. The SD of the SBP (10±3 mmHg) in the patients, over the one-year study period indicated large variations in SBP (Table II).

### Correlation of plasma BNP level with SBP variability and mean SBP

Linear regression analysis was performed to assess the correlation between parameters of SBP variability, mean SBP and plasma BNP levels at the end of the one-year observation period (Table III). Only mean SBP showed a significant correlation (r=0.599, P<0.001) with plasma BNP levels. With regard to the parameters of SBP variability, no significant correlation was observed between plasma BNP levels and the SD or the CV of the SBP. Stepwise multiple regression analysis was performed in order to identify which parameters were independently associated with plasma BNP levels. This revealed that only the mean SBP value (β-coefficient=0.613; P<0.001) was independently associated with plasma BNP levels (Table IV).

## Discussion

The present study compared SBP variability and mean SBP over a one-year observation period in order to identify the association between these variables and plasma BNP levels in patients with hypertension. The highest levels of SBP were observed during December and March, whereas the lowest levels were observed during September. These findings suggest the following: i) SBP variability during the one-year period was not correlated with the plasma BNP levels detected at the end of the observation period; ii) mean SBP during the one-year period was correlated with the plasma BNP levels detected at the end of the observation period and iii) only the mean values of SBP were independently associated with plasma BNP levels.

Seasonal variation in blood pressure is a well-established phenomenon (8–15), with blood pressure being higher during the winter than the summer. In a previous study, we reported that the highest levels of SBP were observed during February and the lowest levels were found during August, in healthy elderly subjects evaluated using home blood-pressure measurements (20). In the present study, the highest levels of SBP were observed in December and March. This represented a slight variation from previous reports (8–15,20). However, in the present study, SBP was detected at an outpatient clinic and included patients who were receiving treatment for hypertension. These differences in methods of blood pressure detection and study participants may be responsible for the discrepancy between the present and previous studies.

The present study aimed to investigate the association between cardiac damage and SBP variability and mean SBP through detecting plasma BNP levels. BNP was found to be correlated with the mean values of SBP, but not with parameters of SBP variability in treated patients with hypertension. These findings indicate that mean values of SBP rather than SBP variability may be a marker of cardiac damage in treated patients with hypertension. Visit-to-visit variability in SBP (SD of SBP) has been demonstrated to be a strong predictor of stroke, independent of mean SBP (18,19). Therefore, further studies may be required in order to determine whether patients with hypertension who demonstrate high visit-to-visit SBP variability have a high risk of cardiac events, including heart failure and ischemic heart diseases.

The specific mechanism through which plasma BNP levels were found to be associated with mean SBP levels, but not SBP variability, is yet to be elucidated. BNP is a hormone secreted by ventricular cardiomyocytes in response to pressure overload of the left ventricle (1). Increases in plasma BNP levels may occur as a consequence of long periods of high blood pressure. Therefore, in the present study, it may be that the patients with hypertension who exhibited SBP variability did not experience persistent high blood pressure. In using plasma BNP levels to assess cardiac damage, the clinical significance of SBP variability and mean SBP may differ.

In conclusion, plasma BNP levels showed stronger correlation with mean values of SBP than with parameters of SBP variability during a one-year observation period. Therefore, the plasma BNP levels taken at the end of the study year may reflect average SBP control, but not the SBP variability during the study year in patients with hypertension.

## Figures and Tables

**Table I:** Clinical parameters in patients with hypertension.

Parameter	Value
Number (male/female)	44 (21/23)
Age (years)	73±9
BMI (kg/m^2^)	24.0±3.9
Diabetes (%)	20
Dyslipidemia (%)	41
Total cholesterol (mg/dl)	190±28
HDL cholesterol (mg/dl)	52±12
Triglycerides (mg/dl)	135±81
BUN (mg/dl)	16.3±4.4
Creatinine (mg/dl)	0.77±0.22
Uric acid (mg/dl)	5.3±1.3
Hb (g/dl)	13.2±1.5
CRP (mg/dl)	0.13±0.16
HbA1c (%)	5.6±0.9
BNP (pg/ml)	39.0±25.7
Drug administration	
ARB (%)	66
CCB (%)	80
α-blocker (%)	3
β-blocker (%)	30
Diuretic (%)	18
Statins (%)	27
Blood pressure and heart rate over one year	
Mean value of SBP (mmHg)	130±13
Mean value of DBP (mmHg)	69±6
Mean value of HR (beats/min)	68±5
SBP variability over one year	
SD of SBP (mmHg)	10±3
CV of SBP (mmHg)	0.08±0.02

Data are presented as mean ± SD unless otherwise indicated. BMI, body mass index; HDL, high-density lipoprotein; BUN, blood urea nitrogen; Hb, hemoglobin; CRP, C-reactive protein; HbA1c, glycosylated hemoglobin; BNP, brain natriuretic peptide; ARB, angiotensin II receptor blocker; CCB, calcium channel blocker; SBP, systolic blood pressure; DBP, diastolic blood pressure; HR, heart rate; SD, standard deviation; CV, coefficient of variation.

**Table II:** SBP variability during one-year observation in patients with hypertension.

Month	SBP (mmHg)
January	132±17
February	134±18
March	135±16
April	128±16
May	130±15
June	130±15
July	128±14
August	130±18
September	126±14
October	130±17
November	132±17
December	135±18

SBP, systolic blood pressure.

**Table III:** Correlation between BNP and other clinical parameters in patients with hypertension.

Parameter	r	P-value
Age	0.388	0.009
BMI	−0.197	0.200
Diabetes	0.052	0.735
Dyslipidemia	0.018	0.907
Total cholesterol	−0.269	0.078
Triglycerides	−0.121	0.434
HDL cholesterol	0.135	0.400
BUN	−0.020	0.896
Creatinine	−0.026	0.865
Uric acid	0.010	0.965
Hb	0.079	0.610
CRP	−0.050	0.745
HbA1c	−0.118	0.450
Drug administration		
ARB	0.002	0.989
CCB	−0.006	0.971
α-blocker	−0.042	0.787
β-blocker	−0.061	0.695
Diuretics	−0.152	0.323
Statins	0.020	0.896
Blood pressure and heart rate		
Mean value of SBP	0.599	<0.001
Mean value of DBP	0.173	0.263
Mean value of HR	0.014	0.930
SBP variability		
SD of SBP	0.219	0.153
CV of SBP	0.058	0.709

BMI, body mass index; HDL, high-density lipoprotein; BUN, blood urea nitrogen; Hb, hemoglobin; CRP, C-reactive protein; HbA1c, glycosylated hemoglobin; ARB, angiotensin II receptor blocker; CCB, calcium channel blocker; SBP, systolic blood pressure; DBP, diastolic blood pressure; HR, heart rate; SD, standard deviation; CV, coefficient of variation.

**Table IV:** Multiple regression analysis of BNP and significantly associated variables.

Independent variable	β-coefficient	t-value	P-value
Mean value of SBP	0.613	4.717	<0.001

F ratio=22.252; r^2^=0.376. SBP, systolic blood pressure.
